# Rectangular Array Electric Current Transducer with Integrated Fluxgate Sensors

**DOI:** 10.3390/s19224964

**Published:** 2019-11-14

**Authors:** Pavel Ripka, Pavel Mlejnek, Pavel Hejda, Andrey Chirtsov, Jan Vyhnánek

**Affiliations:** 1 Department of Measurement, Faculty of Electrical Engineering, Czech Technical University in Prague, 16627 Prague 6, Czech Republic; mlejnp1@fel.cvut.cz (P.M.); chirtand@fel.cvut.cz (A.C.); vyhnajan@fel.cvut.cz (J.V.); 2 Institute of Geophysics of the Czech Academy of Sciences, 14131 Prague 4, Czech Republic; ph@ig.cas.cz

**Keywords:** current sensor, microfluxgate sensors, circular sensor array, finite element modelling

## Abstract

Novel rectangular yokeless current transducer with the range 400 A using 16 microfluxgate sensors around the busbar conductor is presented in this paper. Compared to yokeless transducers utilizing the differential pair of magnetic sensors, our solution has much better suppression of the external currents (lower crosstalk). Compared to industrial transducers with yoke, the new transducer has 15-times lower noise, 7-times better temperature stability, and same crosstalk. Sensor design and design of current monitoring system is presented together with the results of long-term field tests. Crosstalk error is examined in dependence on the number of the operating sensors and external current position.

## 1. Introduction

Small-size and cheap current sensors are required for smart grids, smart buildings and electric drives [1].

Contactless transducers for the electric current usually have a magnetic core or yoke around the current conductor. The purpose of the yoke is to concentrate the magnetic flux inside this yoke because of its small reluctance [2]. Magnetic yoke has significant advantages—the measurement does not depend on the position of the current conductor inside the yoke, and the yoke shields against the external magnetic fields, including high gradient fields from other currents in the vicinity of the transducer.

If only the AC current component is measured, current transformers are the first choice. DC/AC current transducers usually use DC magnetic sensor in the airgap of the yoke. The most popular for this application are Hall sensors. Hall sensors are cheap, they have large range, but they suffer from poor temperature stability of the offset and gain, low linearity and from large noise. By using a microsystem with continuous sensitivity calibration the achievable sensitivity drift is below 80 ppm/°C and the nonlinearity is less than ±0.08%; however, the offset drift is still 300 nT/°C [3]. Better stability can be achieved by using microfluxgate [4], but as the sensor is sensitive to the in-plane field, it should be placed into the circumferential slot.

Transducers with magnetic yoke have several disadvantages such as large size and weight especially in case of large currents. Due to the used magnetic material, cored sensors have poor linearity, remanence, and they can even saturate. The cored sensor accuracy can be increased by using feedback compensation, but for high measured currents this can be power consuming or, in case of pulse currents, even impossible.

Rogowski coils and similar devices are coreless, but they can measure only the AC currents [5]. First approach for measuring the DC/AC current are the transducers without yoke. First type of yokeless DC/AC current transducer uses hole drilled in the middle of the busbar with inserted 2 microfluxgate sensors located on the opposite sides of the PCB [6]. This setup allows us to measure the current with the range ±500 A or even higher and with the linearity error lower than 0.1%. The advantage of this solution is simplicity, low-power consumption and low-cost. The disadvantage of this method is mainly crosstalk effect, which can be eliminated by using a configuration with larger number of microfluxgate sensors; another disadvantage is the need for the drilling hole into the busbar. The other problem is strong frequency dependence due to the non-uniform current distribution caused by the eddy currents in the solid bar. In [7] the busbar with modified shape to improve the frequency characteristics is shown. A similar busbar sensor with a range of 300 A is described in [8]. It uses an AMR sensor bridge in a semi-cylindrical slot in the busbar. Another techniques to improve the frequency characteristics of the busbar sensors is the conductive shielding [9].

Circular optical fiber sensors are based on Ampere law. The fiber measures the line integral of the magnetic field, which is proportional to the sum of currents surrounded by the line. This means that the reading does not depend on the conductor position within the fiber loop, nor on the external current or magnetic field. The first type of this sensor is based on magnetooptical Faraday effect (Delgado et al. 2016), second type is based on magnetostriction of the nickel cover of the fiber [10]. The disadvantage of both fiber sensor types is low sensitivity and large drift.

Circular sensor arrays are becoming popular due to their ability to suppress the influence of the conductor position and their good crossfield immunity [11]. These arrays are also based on the Ampere law and approximate the field integral by a sum of point sensor reading. This approach was recently used in [12] using microfluxgate sensors and also Hall sensors [13]. The shape of the circular array is rather impractical for large current bars. The measuring setup with AMR sensors has only limited range (typically ±8 A) due to the fact that the precise AMRs have limited field range of only 200 µT. However, in principle the measuring range of AMR sensors can be up to 6 mT [14]. GMR and TMR sensors are not suitable for this application, as they do not provide sufficient linearity and suffer from hysteresis. Magnetoelectric sensors do not have stability required for DC/AC current sensors [15].

In this paper we describe a novel rectangular yokeless current transducer using microfluxgate sensors. This concept was first introduced in [16]. We compare the performance of our new transducer with the commercially available sensor HOP 800-SB by LEM, which is based on the uncompensated Hall sensor in the airgap of the rectangular yoke. The comparison in this paper is based on the laboratory tests as well as the long-time field use.

## 2. Yokeless Transducer Design

**Figure 1:**
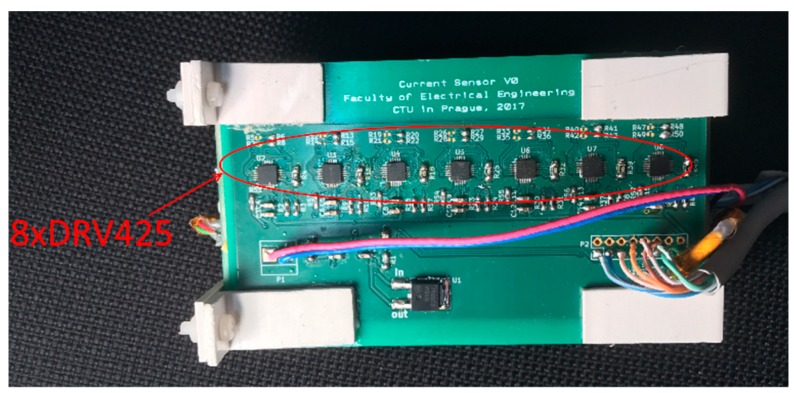
Busbar current transducer using 16 microfluxgate sensors around the measured current. The top 8 sensors are visible on this photograph.

**Figure 2:**
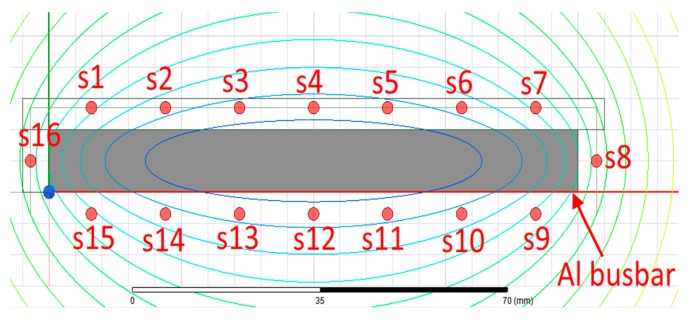
Distribution of the microfluxgate sensors around the busbar conductor.

In this paper, we suggest a new transducer for measuring the DC/AC current flowing through the massive busbar. We use 16 integrated microfluxgate sensors TI DRV425 placed around the conductor. The sensors are mounted by soldering to the printed circuit board. The transducer is designed for maximum current of 400 A. Fluxgate sensors are much more sensitive than Hall sensors and they have better temperature stability, noise and non-linearity. The disadvantage compared to Hall sensors is their relatively small full range of 2 mT. Our transducer is designed to measure DC and AC current in the aluminum busbar conductor with the cross-section of 100 × 10 mm which is commonly used as the ground conductor in power stations. The transducer has dimensions of 110 × 20 × 63 mm (aperture: 104 × 14 mm) which makes it very compact and easy to install. Each microsensor is individually feedback compensated. All the excitation and signal processing electronics is integrated inside the sensor chips, the only adjustable external component is a shunt resistor. The full-scale range of the microsensor and, therefore, the range of the current transducer can be adjusted using the shunt resistors of individual sensors. Utilizing such a number of operating sensors can significantly reduce the influence of the external magnetic fields including those caused by external currents by better approximation of the closed line integral in the Ampere’s law. The finite element modelling is performed for evaluation of the design parameters of the sensor and the verification of the measurements. The experimental model of the sensor is shown in Figure 1. Commercially available LEM sensor HOP 800-SB is used to compare the properties of the yokeless current transducer. The LEM sensor consists of 2 Hall sensors in the airgaps of the magnetic core and operates without any feedback compensation. Distance between the particular integrated fluxgate sensors DRV425 is 14 mm and the distance between the sensors and the surface of the busbar is 3.5 mm. Distribution of the microfluxgate sensors around the busbar is shown in Figure 2. The sensor outputs are read by the NI-DAQ card and processed by the software.

## 3. Signal Processing

Each sensor should have individual weight for the correct computation of the current. The FEM simulation is performed to find the weights. For the material properties are the following parameters: for the aluminum relative permeability µ = 1.000021 and conductivity S = 31.713 × 10^6^ S/m. Electrical conductivity of the aluminum busbar was measured using the 4—terminal method with the measuring current of 50.25 A in the region of the homogenous current density. Three different methods of calculating the weight are used for each sensor. The sum of the sensor output signals is an approximation of the Ampere’s law

(1) $$I=\oint_{s}^{}H\;ds$$

### 3.1. Same Weights

All sensors have the same weight w = 0.0147 A/(A/m). The measured current is calculated by the Equation (3).

(2) $$w=w_{1}=w_{2}=w_{3}=\ldots =w_{16}$$

(3) $$\;I_{measured\;}=w\left(H_{1}+H_{2}+H_{3}+\ldots +H_{16}\right)\;$$

### 3.2. Integral Method

The tangential component of the magnetic field strength is calculated by FEM along the line at a distance 3.5 mm from the surface of the busbar for the unit measured current in the busbar. This line is divided into separate regions corresponding to each of 16 sensors. For calculating the weights for each sensor, the line integral corresponding of each section is calculated and divided by the magnetic field strength obtained by FEM for each sensor location.

(4) $$w_{i}=\frac{\int H_{t_{i}}dl}{H_{FEM}i}$$

(5) $$\;I_{measured}=\left(w_{1}H_{1}+w_{2}H_{2}+w_{3}H_{3}+\ldots +w_{16}H_{16}\right)$$

The measured current is calculated by the Equation (5).

### 3.3. Weighted Method

The weight for each sensor was obtained by dividing the current used in the simulation by the calculated value of the tangential magnetic field strength at the location of each corresponding sensor. The equation for calculating the weights for each sensor and measured current is shown in Equations (6) and (7), respectively.

(6) $$w_{i}=\frac{I_{total_{simulation}}}{H_{FEM}{}_{i}}$$

(7) $$\;I_{measured=}=\frac{w_{1}H_{1}+w_{2}H_{2}+w_{3}H_{3}+\ldots +w_{16}H_{16}}{16}$$

## 4. Laboratory Tests

During laboratory tests our new transducer was compared with the commercially available LEM transducer HOP 800-SB which has similar size of the opening for the measured current. The LEM transducer has of 2 Hall sensors in the airgap of the magnetic core and it operates without feedback compensation.

### 4.1. Offset Stability with Temperature

**Figure 3:**
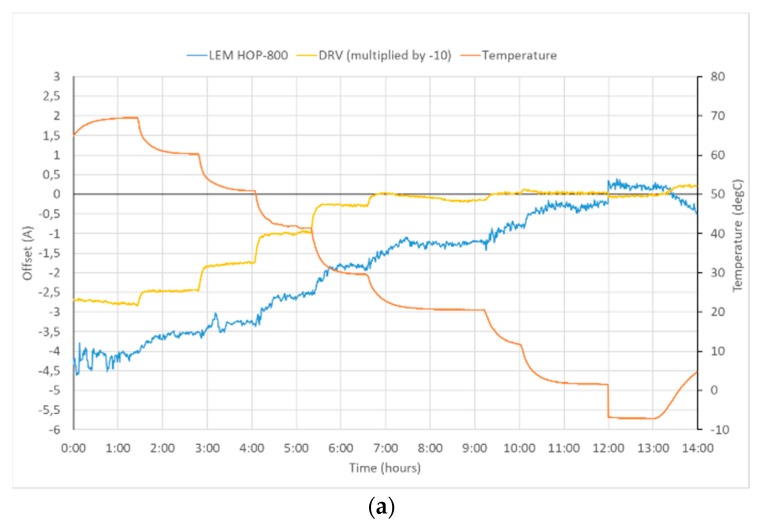

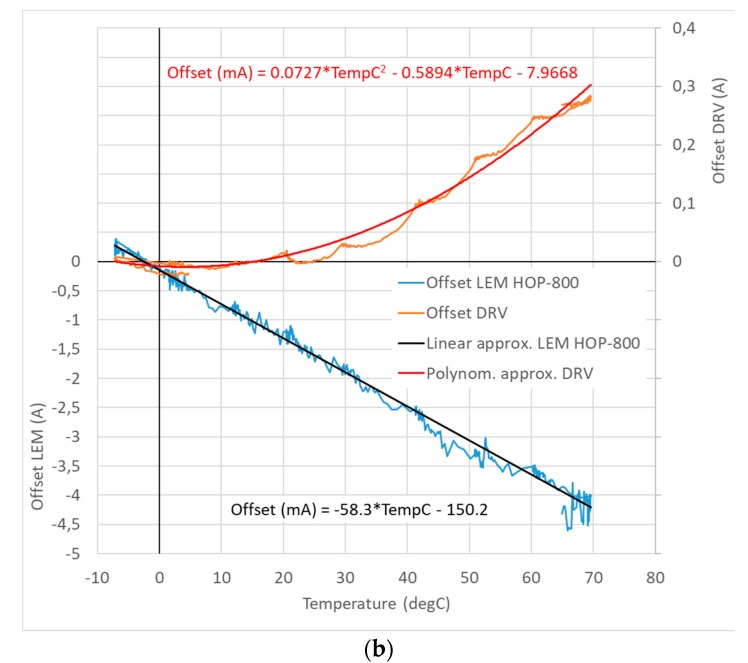
Temperature dependence of the new “DRV” current transducer and LEM HOP 800-SB: (**a**) time plot of temperature and both offsets (**b**) Offset as a function of temperature. Notice 10-times larger scale for LEM sensor for both figures.

Offset drift was tested in the temperature range from −10 °C to +70 °C, which is the operation range of the LEM transducer. The offset of both sensors during the test is shown in Figure 3. While the LEM sensor has shown systematic temperature drift of 58 mA/°C, the offset of our sensor remained within 300 mA range for the whole temperature range (the offset is multiplied by −10 in Figure 3 for better comparison with offset of LEM HOP 800-SB sensor). The temperature offset drift of our sensor was changing between 8 mA/°C at 60 °C to −2 mA/°C between −10 °C and 0 °C.

### 4.2. Noise

**Figure 4:**
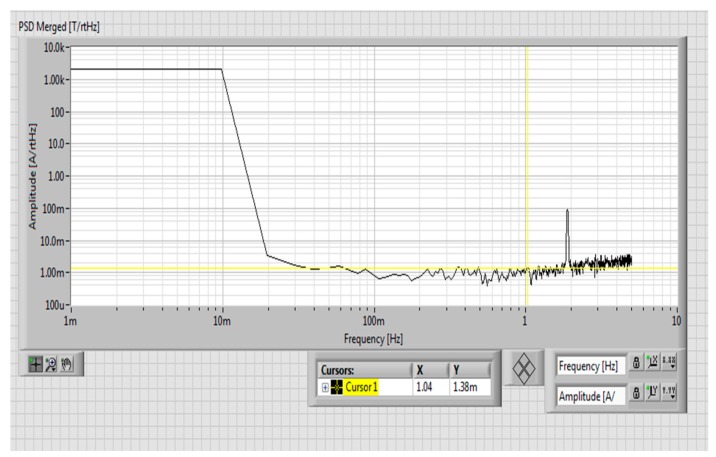
Power spectrum of the data acquisition system for our yokeless current transducer (magnetic sensors were replaced by equivalent dummy resistors).

**Figure 5:**
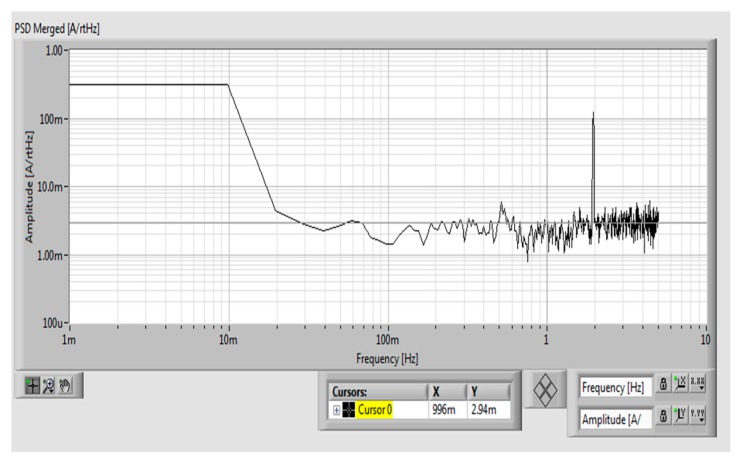
Power spectrum of the complete yokeless current transducer.

The noise spectrum of the novel yokeless transducer was calculated by using the LabVIEW software. The measured noise power spectrum density (PSD) is composed of two main components: the noise of the DAQ card and the noise of the transducer. PSD of the DAQ card itself equals to 1.38 mArms/√Hz at 1 Hz as shown in Figure 4, while total PSD (DAQ card + transducer) is 2.94 mArms/√Hz at 1 Hz as shown in Figure 5. From this we can estimate the noise of the transducer as √(2.94^2^ − 1.38^2^) = 2.6 mArms/√Hz at 1 Hz.

We can compare this noise to the theoretical value calculated from the own noise of microfluxgate sensors: the DRV425 noise is 4 nT/√Hz at 1 Hz, for the sum of 16 sensors the resulting noise is 4*√16 = 16 nT ≈ 12.8 mA/m. Using the current sensitivity w = 0.0147 A/(A/m) we receive the theoretical noise of 0.18 mArms/√Hz at 1 Hz.

**Figure 6:**
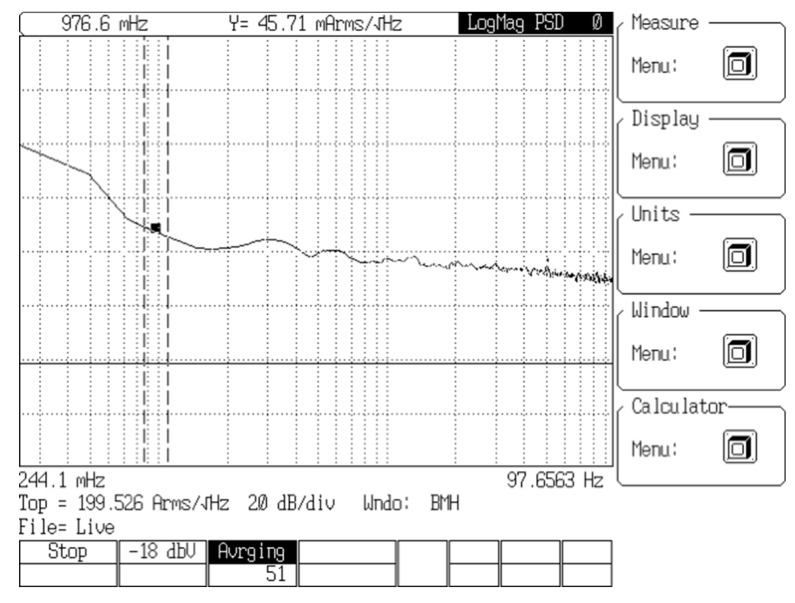
Power spectrum of the LEM HOP 800-SB sensor.

The noise analysis for LEM transducer is performed by the FFT Analyzer SR770. The noise power spectrum is shown in Figure 6. The noise PSD is 45 mArms/√Hz, which is 15-times higher than the noise of the novel yokeless transducer.

We may conclude that the noise of our transducer is 15-times lower compared to the LEM sensor and it is mainly caused by the system noise and interference rather than by the own magnetic noise of the used microfluxgate sensors.

### 4.3. Crosstalk Error of the Reading

**Figure 7:**
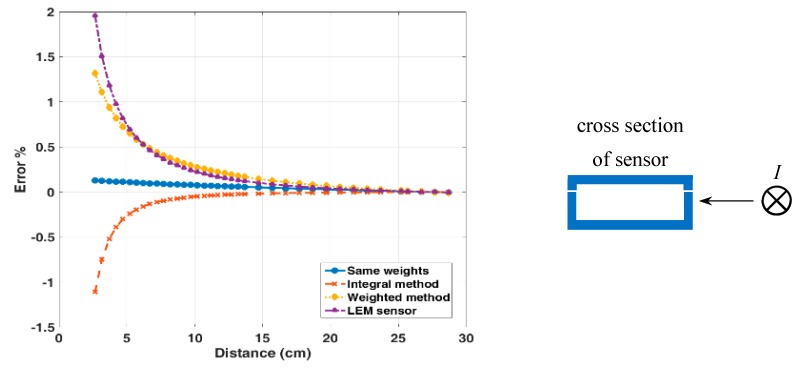
The LEM and the yokeless sensor: influence of the lateral external DC current of 10 A as a function of the distance.

**Figure 8:**
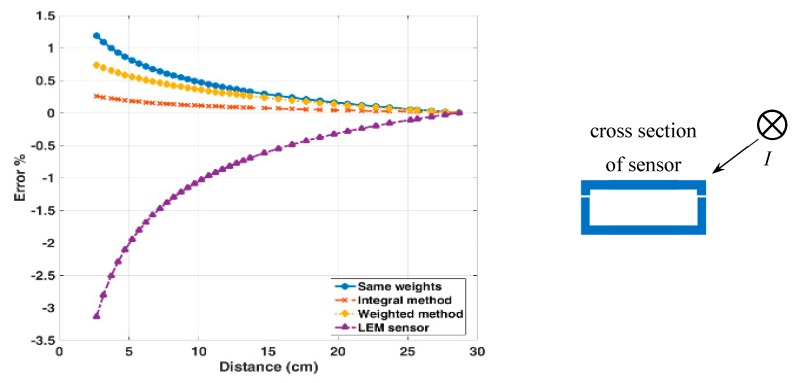
The LEM and the yokeless sensor: influence of the superior external DC current of 10 A as a function of the distance.

**Figure 9:**
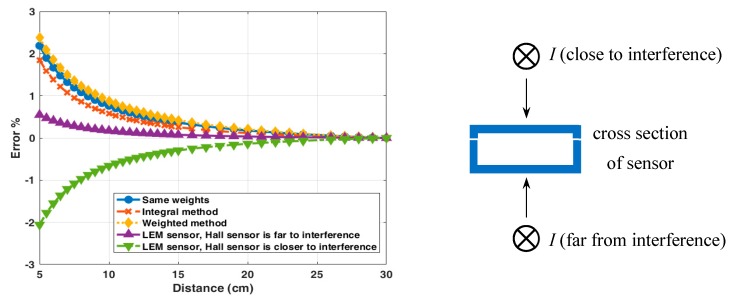
The LEM and the yokeless sensor: influence of the external superior and inferior DC current of 10 A as a function of the distance.

The external current has the significant influence on the current reading of both transducers. This error is known as the crosstalk. We have tested the crosstalk for the currents through the long external conductor parallel with the measured conductor. The measured response to the realistic external in-plane (0° direction) current bar with the DC current of 10 A for our sensor and LEM transducer is measured and shown in Figure 7. The response to the external current in the 45° direction and in 90° direction is also shown in Figure 8 and Figure 9, respectively. The positions of the external current are indicated in the respective figures. The influence of airgaps in the LEM sensor is clearly visible in Figure 9: the external current in the vicinity of the airgap can more easily penetrate into the magnetic circuit and influence the sensor reading. For our yokeless sensor, the crosstalk is caused by the following factors: (1) Finite number of sensors; (2) variations in sensor sensitivities; (3) errors in the position and the sensing direction of individual sensors. For both transducers the crosstalk error depends on the direction of the external current with respect to the sensor.

In the minimum realistic distance of 15 cm in power installations the error of yokeless sensor is always below 0.5%, while for the LEM sensor the same error is 1%.

### 4.4. The Crosstalk Error as a Function of the Number of Sensors

**Figure 10:**
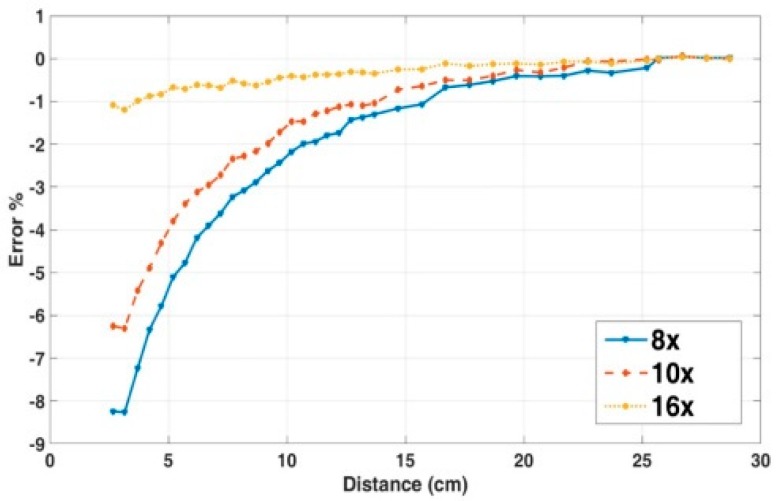
Dependence on the number of the operating sensors, influence of the external DC current in superior position, Same weights method.

As mentioned above, the higher number of the integral points reduces the current reading error. The crosstalk error in dependence on the number of operating microsensors is shown in Figure 10 for the same weights method; for the other method this dependence is very similar. The number of the sensors depends on the application requirements—how accurately and reliable the current should be measured. The current reading error is lowered since the larger number of the sensors allows us to better approximate the integral with an increase in the number of operating sensors.

## 5. Ground Current Monitoring Station

### 5.1. Architecture of the Hardware and Software

**Figure 11:**
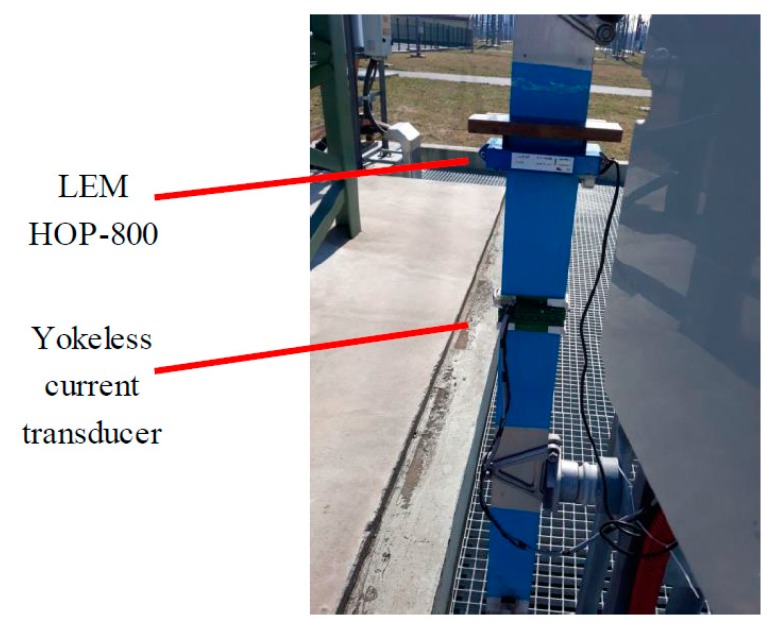
Installation of the current sensors on the measured busbar.

**Figure 12:**
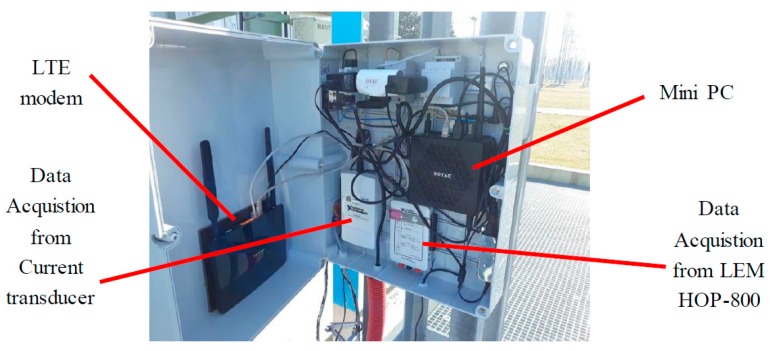
Data acquisition system.

The autonomous measurement system for the current sensing in the neutral line of distribution transformers was designed (see Figure 11). The system consists of the mini PC Zotac ZBOX Nano with two data acquisition devices—USB-6210 and myDAQ, both from National Instruments (see Figure 12). The USB-6210 device acquires the data from all sixteen microfluxgates individually and myDAQ acquires the LEM sensor output. The digital data are online processed in the special software package developed in LabVIEW. All the measured and calculated data are stored on the SSD.

The whole system is connected to the internet via the LTE modem. This connection allows the remote control of the system and reading data. Every day the system sends the email with the aggregated data in the picture for quick overview.

**Figure 13:**
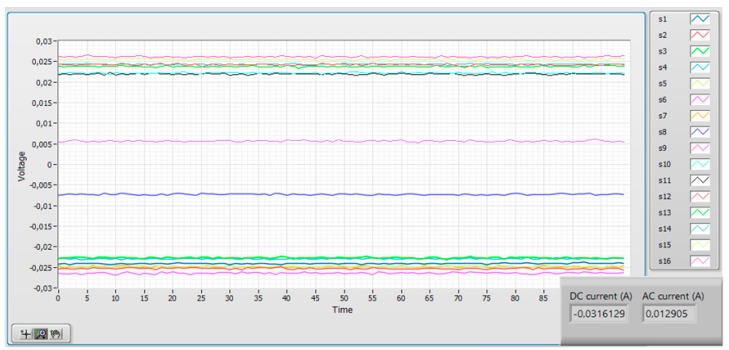
The time plot of the individual microfluxgate sensors from the circular electric current transducer without interference. The DC component is caused by the Earth’s field projection into the sensing axis plus the (slowly changing) sensor offset.

**Figure 14:**
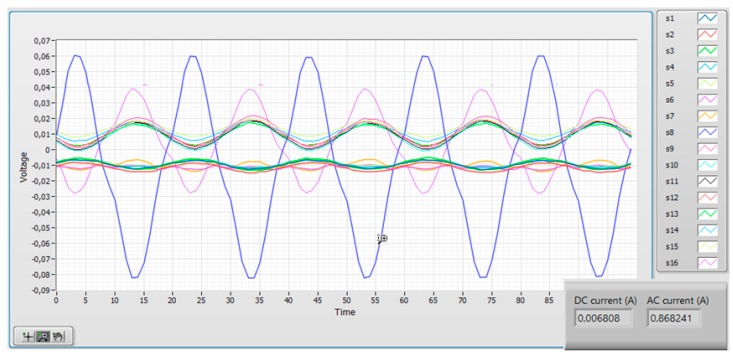
The time plot of the microfluxgate sensors in the noisy industrial environment. The intereference is reduced by averaging all sensors.

The software allows to register the reading of every individual sensor which is important for the transducer diagnostics. Figure 13 shows such record for zero measured current: the DC level of the individual sensors is given mainly by the Earth’s field component in its sensing direction. The slow sensor drift shows good long-term offset stability. Figure 14 shows the time record of individual sensors in the noisy environment: the total current reading shows how the external interference is effectively suppressed.

### 5.2. Long-Term Measurements

**Figure 15:**
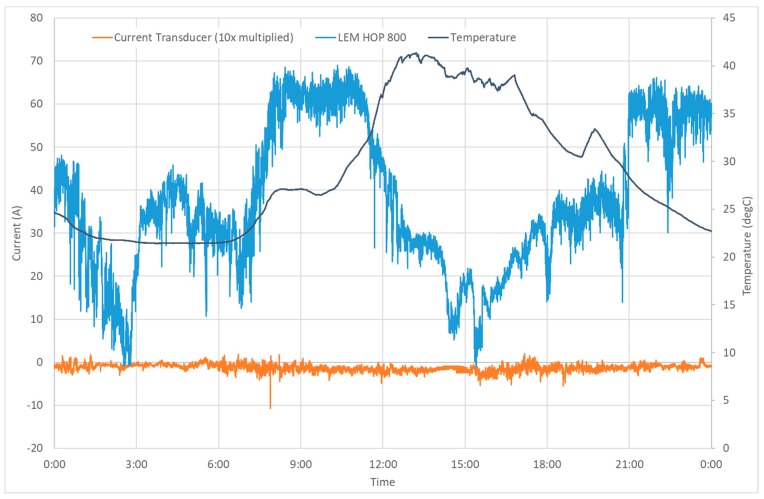
DC ground current and temperature during one day (21 June 2019).

**Figure 16:**
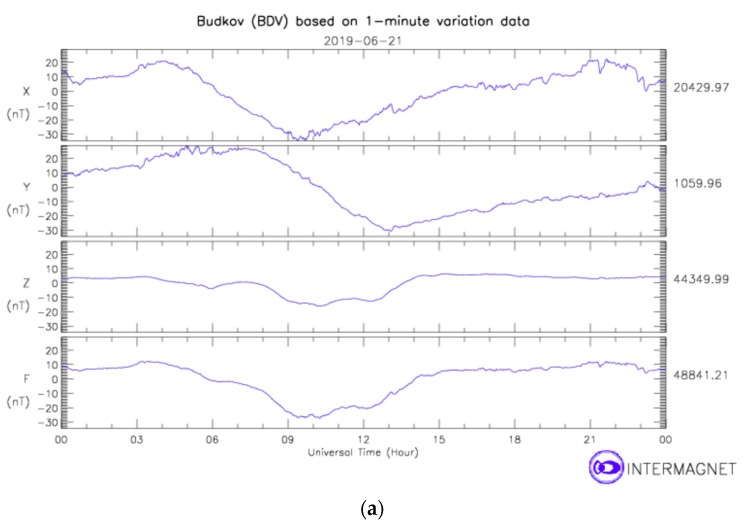

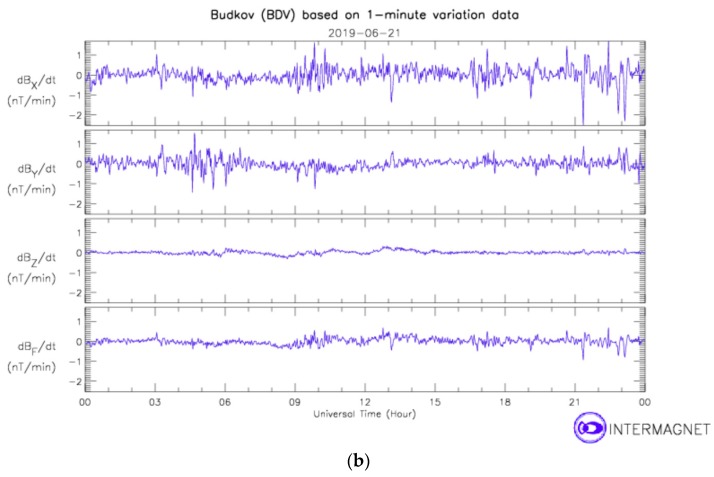
Variation of the Earth’s magnetic field measured at the nearest magnetic observatory variation station Budkov on 21 June 2019: (**a**) x (North), x (West) and z (vertical) components, (**b**) the rate of change. http://www.intermagnet.org.

Our measurement system was installed on the neutral line (see Figure 11) of the distribution transformer located in near Havlickuv Brod (GPS: N 49°34.916′, E 15°36.062′). The location was selected to monitor geomagnetically induced currents. Here we show the current plots on geomagnetically calm days to show the system stability. Figure 15 shows the plot of the transducer outputs and the temperature measured inside the LEM sensor during the magnetically quiet day. The record contains also transient currents of various origin occurring in the grid. Figure 15 confirms that the newly developed sensor has much better stability (the output is multiplied by 10) not only during the laboratory tests, but also at the field installation. Figure 16 shows the Earth’s magnetic field record measured at the nearest variation station Budkov. The magnetic field changes are very small: Only a regular diurnal change with 50 nT amplitude is visible; the geomagnetically induced variations depend on the rate of change of the geomagnetic field which is shown in Figure 16b for the same day. The maximum rate of change was 2 nT/min, while during the magnetic storms this value can be as large as 100 nT/min.

## 6. Conclusions

A current through the busbar can be measured by an array of the integrated fluxgate sensors, resolution of 1 mA is achievable. Using the multisensory array significantly reduces the crosstalk: with 16 sensors the crosstalk error for the external current in the 15 cm distance is below 0.2% for the integral method. The maximum measured current for the given geometry is 400 A, and this range can be increased only by increasing the distance of the sensors from the busbar, which would increase the size of the overall transducer. Our sensor has the noise level of 3 mArms/√Hz at 1 Hz, which is 15-times lower than the industry standard LEM transducer. The temperature drift is max. 8 mA/°C, which is 7-times better than the industrial standard. While the crosstalk error is the same, our sensor is significantly cheaper. The disadvantages of our sensor are higher complexity and power consumption of the multi-sensor system: the peak power consumption is 0.75 W. Long-term testing proved the reliability and stability of the new transducer.

## References

[1] Crescentini M., Marchesi M., Romani A., Tartagni M., Traverso P.A. (2018). A Broadband, On-Chip Sensor Based on Hall Effect for Current Measurements in Smart Power Circuits. IEEE Trans. Instrum. Meas..

[2] Ripka P. (2019). Contactless measurement of electric current using magnetic sensors. Tech. Mess..

[3] Ajbl A., Pastre M., Kayal M. (2013). A Fully Integrated Hall Sensor Microsystem for Contactless Current Measurement. IEEE Sens. J..

[4] Lee D.W., Eissa M., Gabrys A., Shulver B., Mazotti E., Lavangkul S., Chevacharoenkul S., Murphy N., Wang F.C., Zhang Y.S. (2017). Fabrication and Performance of Integrated Fluxgate for Current Sensing Applications. IEEE Trans. Magn..

[5] Wang J.G., Si D.C., Tian T., Ren R. (2017). Design and Experimental Study of a Current Transformer with a Stacked PCB Based on B-Dot. Sensors.

[6] Snoeij M.F., Schaffer V., Udayashankar S., Ivanov M.V. (2016). Integrated Fluxgate Magnetometer for Use in Isolated Current Sensing. IEEE J. Solid-State Circuits.

[7] Ripka P., Grim V., Petrucha V. (2017). A Busbar Current Sensor With Frequency Compensation. IEEE Trans. Magn..

[8] Zhang Z.H., Syuji O., Osamu A., Hideto K. (2015). Development of the Highly Precise Magnetic Current Sensor Module of +/-300 A Utilizing AMR Element With Bias-Magnet. IEEE Trans. Magn..

[9] Blagojevic M., Jovanovic U., Jovanovic I., Mancic D., Popovic R.S. (2016). Realization and optimization of bus bar current transducers based on Hall effect sensors. Meas. Sci. Technol..

[10] Moron C., Cabrera C., Moron A., Garcia A., Gonzalez M. (2015). Magnetic Sensors Based on Amorphous Ferromagnetic Materials: A Review. Sensors.

[11] Yu H., Qian Z., Liu H.Y., Qu J.Q. (2018). Circular Array of Magnetic Sensors for Current Measurement: Analysis for Error Caused by Position of Conductor. Sensors.

[12] Weiss R., Makuch R., Itzke A., Weigel R. (2017). Crosstalk in Circular Arrays of Magnetic Sensors for Current Measurement. IEEE Trans. Ind. Electron..

[13] Itzke A., Weiss R., Weigel R. (2019). Influence of the Conductor Position on a Circular Array of Hall Sensors for Current Measurement. IEEE Trans. Ind. Electron..

[14] Jogschies L., Klaas D., Kruppe R., Rittinger J., Taptimthong P., Wienecke A., Rissing L., Wurz M.C. (2015). Recent Developments of Magnetoresistive Sensors for Industrial Applications. Sensors.

[15] Zhang M.J., Or S.W. (2018). Gradient-Type Magnetoelectric Current Sensor with Strong Multisource Noise Suppression. Sensors.

[16] Chirtsov A., Ripka P., Vyhnanek J. (2018). Rectangular Array Current Transducer with Integrated Microfluxgate Sensors. IEEE Sens. Conf..

